# The Co-Management of Tuberculosis and Diabetes: Challenges and Opportunities in the Developing World

**DOI:** 10.1371/journal.pmed.1001269

**Published:** 2012-07-24

**Authors:** Timothy Sullivan, Yanis Ben Amor

**Affiliations:** 1 Department of Medicine, Mount Sinai Hospital, New York, New York, United States of America; 2 The Earth Institute, Columbia University, New York, New York, United States of America

## Abstract

Yanis Ben Amor and Timothy Sullivan call for more research to examine whether the diagnosis and treatment of diabetes in TB patients may improve TB outcomes and patient survival.

**Summary Points**

- The evidence for a link between tuberculosis (TB) and diabetes is strong. Patients with diabetes are at increased risk of developing active TB, and have higher rates of treatment failure and death, even when placed on appropriate TB treatment.
- This link may become even more meaningful in coming years, as the prevalence of diabetes is expected to rise dramatically in the resource-poor areas where TB thrives.
- We discuss some of the financial challenges inherent in treating diabetes in patients with TB, as well as some opportunities for cost-saving and collaboration in the co-management of these two diseases.
- We also propose strategies for funding and implementing efforts to control TB and diabetes concurrently in the developing world, and highlight areas of much-needed research in this field.

## Introduction

Tuberculosis (TB) kills more than 3,500 people each day worldwide, leading to approximately 1.4 million deaths every year [1]. One-third of the world's population is currently infected with the causative agent of TB, and 8.8 million new cases of active TB are estimated to occur around the world each year [1]. TB is fueled by several social and economic factors, such as poverty or malnutrition [2], as well as other infectious diseases, such as HIV. People living with HIV/AIDS are 21–34 times more likely to develop active TB [1], and this harmful synergy has led public health systems to attempt to tackle the two diseases concurrently.

In recent years, strong evidence has been gathered to confirm a link between TB and yet another disease: diabetes mellitus. That link had been suspected for centuries [3]. Many studies now show that diabetes may be associated with an increased risk of developing active TB [4]–[6], and that TB patients who also have diabetes may have higher rates of treatment failure and death [7],[8]. These findings have led some to wonder if the diagnosis and treatment of diabetes in TB patients may improve TB outcomes. More research into this question is clearly needed [9],[10]. However, if further studies show that treating diabetes can improve TB outcomes, then should existing TB funding and treatment mechanisms in place in the developing world be expanded to include diabetes care? How could the existing funding and control efforts aimed at a mostly curable infectious disease be adapted to manage a chronic, non-communicable condition?

## TB and Diabetes

Several recent publications have described the association between diabetes and TB, specifically the increased prevalence of active TB among patients with diabetes and the poorer treatment outcomes in these patients when compared to those without diabetes [11],[12]. For example, one recent systematic review of 13 studies reported that diabetic patients had about a 3-fold increased risk of developing TB when compared to those without diabetes [5].

Others have reported the poorer treatment outcomes in patients with diabetes and TB, including a higher risk of death among these patients. One prospective study examined sputum cultures at the completion of 6 months of TB treatment, finding positive cultures in 22.2% of patients with diabetes and 6.9% of those without diabetes [8]. Another systematic review of treatment outcomes described a relative risk of death of 1.89 among TB patients with diabetes when compared to non-diabetic patients [12]. After controlling for potential confounders, the pooled risk of death among TB patients with diabetes was nearly five times greater than those without diabetes [12].

The link between these two diseases may become even more meaningful in coming years, as the prevalence of obesity and diabetes are expected to rise dramatically in the resource-poor areas where TB thrives [13]. In sub-Saharan Africa, for example, the number of patients with diabetes is projected to increase by 161% between 2000 and 2030 [14].

Some groups have suggested that the burden of diabetes in these countries may hinder progress towards attaining the United Nations' (UN) Millennium Development Goal (MDG) and specific targets related to TB control [4],[15]. One study showed that in a southern Mexican population the impact of diabetes on the incidence of TB was greater than that of HIV [16]. This close relationship between TB and diabetes raises the question of whether diabetes treatment and TB care should become more integrated, similar to recent efforts to link the management of TB and HIV. For example, the Stop TB Partnership's Global Plan to Stop TB has called for greater collaboration between TB and HIV programs at multiple levels, and has estimated that US$2.8 billion would be required to fund TB-HIV co-infection programs between 2011 and 2015 [17].

In 2011, the World Health Organization (WHO) and The International Union Against Tuberculosis and Lung Disease released a report acknowledging the association between TB and diabetes and similarly calling for increased collaboration between TB and diabetes control efforts [9]. In addition to providing provisional suggestions for implementing these changes, this report highlights the lack of evidence to guide such work and advocates for more research into the efficacy and cost-effectiveness of any collaborative efforts.

Although numerous etiological risk factors affect TB incidence and treatment outcomes, some, such as poverty, malnutrition, or indoor air pollution, would be exceedingly difficult for existing health systems or TB control programs to address for logistical reasons. Diabetes and HIV are similar in that treating these illnesses presents an opportunity for feasible collaboration with current TB control efforts, if sufficient funding can be found.

## Funding Challenges

A major impediment to expanding diabetes care in the developing world is the high cost. One study in Tanzania showed that the monthly cost of insulin treatment represents 25% of the minimum wage [18], while another showed that the yearly cost of diabetes treatment with insulin amounted to 75% of the per capita income in Mozambique and 61% in Mali [19]. In addition to the expenses linked to insulin and oral hypoglycemic medications, the direct costs of diabetes care include outpatient visits, inpatient admissions, and the management of the micro- and macrovascular complications of this disease. While these direct costs vary according to country, they were estimated at US$138 per patient per year in Tanzania [20], and US$149 per patient per year in India [21], demonstrating a stark contrast with the mean health expenditures per person with diabetes in 2010, estimated at US$112 in sub-Saharan Africa (US$30 in Tanzania) and US$53 in South East Asia (US$55 in India) [22]. Access to diabetes services also varies greatly among developing countries, where limited health care systems are often designed to address acute, not chronic, illnesses [23]–[25]. Although the UN and other international organizations are demonstrating increasing interest in addressing non-communicable diseases (NCDs) such as diabetes, clearly more funding will be needed [26].

One option might be for the organizations that fund global TB control efforts to expand their missions to include diabetes care. These organizations already have a robust fundraising and operational infrastructure in place that could help to rapidly expand diabetes treatment in developing countries.

To calculate the amount of funding that these organizations would need to allocate to treat diabetes in TB patients in developing countries, we used the estimated direct costs of diabetes treatment in Tanzania and India and figures from a study assessing the yield of finding additional diabetes cases through systematic screening of patients with active TB [27]. We estimated the cost of treating all additional diabetes cases detected in TB patients for 6 months, which is the duration of Directly Observed Therapy – Short Course (DOTS). This cost was calculated based on the estimated TB incidence (Table 1) and the number of actual TB cases detected (Table 2) in the two WHO-defined regions with the highest burden of TB, South East Asia and Africa.

**Table 1:** Additional funding required for diabetes care in TB patients using estimated TB incidence in Africa and South East Asia.

WHO Region	Estimated TB Incidence (2010) in Millions^a^	Additional Diabetes Cases Diagnosed through Screening^b^	Cost of Treating Diabetes for the Duration of DOTS in $US Million^c^
AFR^d^	2,3	[43,700–805,000]	[3–55.5]
SEA^e^	3,5	[66,500–1,225,000]	[5–91.9]

a As reported in the 2011 WHO Global Tuberculosis Control report [1].

b Using a yield of additional cases of diabetes per active TB case screened ranging from 1.9% to 35% [27].

c Using as an approximation of direct cost of diabetes care per patient per year a figure of $138 for sub-Saharan Africa (based on estimates from Tanzania [20]) and of US$149 for South East Asia (based on estimates from India [21]), for a duration of 6 months.

d Sub-Saharan African region.

e South East Asian region.

**Table 2:** Additional funding required for diabetes care in TB patients using actual number of TB cases diagnosed in Africa and South East Asia.

WHO Region	Actual Cases Diagnosed (2010) in Millions^a^	Additional Diabetes Cases Diagnosed through Screening^b^	Cost of Treating Diabetes for the Duration of DOTS in $US Million^c^
AFR^d^	1,38	[26,220–483,000]	[1.8–33.3]
SEA^e^	2,14	[40,565–747,250]	[3–56]

a As reported in the 2011 WHO Global Tuberculosis Control report [1].

b Using a yield of additional cases of diabetes per active TB case screened ranging from 1.9% to 35% [27].

c Using as an approximation of direct cost of diabetes care per patient per year a figure of $138 for sub-Saharan Africa (based on estimates from Tanzania [20]) and of US$149 for South East Asia (based on estimates from India [21]), for a duration of 6 months.

d Sub-Saharan African Region.

e South East Asian Region.

Using the estimated TB incidence (Table 1), the additional funding required for diabetes care ranged from US$3 million to US$55 million per year in Africa and from US$5 million to US$92 million per year in South East Asia. Based on actual numbers of TB cases detected (Table 2), we have estimated the additional funding for diabetes care to be US$2–33 million per year in Africa and US$3–56 million per year in South East Asia. The range in funding directly reflects the range in additional yield of diabetes cases, which was shown to vary from 1.9% to 35% [27].

As a point of comparison, the WHO reported the funding from domestic and donor sources for the 97 countries with 92% of the world's TB cases to amount to US$4.4 billion in 2012 (the funding gap reported by countries amounted to close to US$1 billion in 2012) [1], of which the contribution of the Global Fund to Fight AIDS, TB and Malaria accounts for 12% (or 82% of all international funding). The Global Fund has already provided US$3.2 billion in 112 countries to help fight TB. New grants allocated by the Global Fund might temporarily be in jeopardy due to its current financial situation [28]. If that predicament is solved, future TB-specific grants could include a diabetes co-management component to increase the score of TB proposals received. Similarly, the Global Plan to Stop TB has called for US$67 billion over 10 years to reduce the global burden of the disease, part of which could be specifically allocated to the co-management of the two diseases [17]. Whether these organizations will eventually help finance diabetes care in the future will ultimately be determined not only by the missions of these funds and their financial state, but also the expected benefit and cost-effectiveness of any new intervention. More research is surely needed to help inform these decisions.

Expanding TB treatment to include diabetes care would also create some foreseeable questions and challenges. Importantly, should the TB community pursue diabetes primary prevention in patients without active TB, since diabetes is clearly a risk factor for active TB, or should the focus be on treating diabetes in those who develop TB? And, if diabetes treatment is incorporated with TB care, who would fund the long-term management of diabetes in these patients after the TB is treated? Considering the current funding challenges, the co-management of the two diseases could initially be limited to patients with active TB while they are being treated for their TB, which served as reference for our funding estimates.

## Opportunity for Collaboration

Although expanding TB treatment to include diabetes care would be expensive, perhaps existing health systems used for TB could be adapted to diabetes management, which may help control costs. For example, some have argued that DOTS, the framework already in place for TB control in many developing countries, could be modified to help with the management of NCDs like diabetes [29]. Although DOTS was originally designed to deliver a short course of TB treatment, a recent review has assessed that directly observed therapy can also be effective in the chronic management of HIV, particularly when targeting non-adherent patients [30]. Given that diabetes was shown to be effectively managed by non-physician clinicians in developing countries [31],[32], community health workers already in place to deliver TB care could be further trained to help manage diabetes as well.

In light of recent calls for increasing the presence of community health workers throughout the developing world [33], the training and implementation of this workforce could be another opportunity for those engaged in TB, HIV, and NCD efforts to work together. Future generations of community health workers could therefore be trained to manage both acute and chronic illnesses concurrently.

Another pillar of the DOTS framework is the creation of systems to insure a steady supply of TB medications. Diabetes patients would certainly benefit from the same logistics and commitment, as the supply of insulin has been shown to be unreliable in poor countries [34].

Yet another avenue for potential collaboration between TB and diabetes efforts is in the promotion of effective and affordable diagnostic testing. The DOTS framework includes a commitment to improving laboratory support, and the strengthening of laboratory systems has been identified as a major priority within the Global Plan to Stop TB [17]. Similarly, diagnostic testing for diabetes, including simple point-of-care blood glucose testing, is still lacking in many developing nations. A 2005 study reported that only 6% of health centers surveyed in Mozambique and 25% in Zambia were capable of testing blood glucose levels [35]. Although TB and diabetes diagnostics rely on very different technology, improving basic lab services in developing countries would undoubtedly benefit the diagnosis and management of both illnesses.

### Our Proposed Strategy for Co-Management of TB and Diabetes

If the link between effective control of diabetes at the onset of TB treatment and improved outcomes among TB patients is clearly demonstrated by further studies, we propose that all confirmed TB patients be systematically screened for diabetes, and that all diabetic patients be screened for TB when symptomatic.

Co-afflicted patients without existing diabetes care should subsequently be managed by the relevant local or national TB program under DOTS for at least the duration of TB treatment to ensure proper and continuous management of both diseases. Although it would also be feasible to refer these patients to existing diabetes programs, these programs lack sufficient funding and infrastructure in many developing countries [25].

Expanding local TB programs to include diabetes care would necessitate the training of community health workers in basic diabetes care, such as blood glucose testing and medication management, as well as the consultation of nurses and physicians trained in diabetes management.

Following confirmed cure or completion of TB treatment, two options for ongoing diabetes care would be offered depending on the level of funding available to the TB control program:

Diabetic patients could be transferred to the diabetes control program, in countries where such programs exist, for continued management of their condition. As former TB patients, with a higher risk of relapse compared to non-diabetic patients, they would be required to return for regular TB screenings;
Diabetic patients would remain part of the DOTS structure after completion of TB treatment to ensure optimal follow-up and rapid detection of possible relapses.

The TB control programs should also develop educational materials to be distributed at diabetes treatment centers, to inform diabetic patients of their risk of developing active TB. More specifically, symptoms of TB (cough, fever, night sweats, weight loss, and chest pain) should be clearly highlighted to advise diabetic patients when to seek TB screening.

## Looking Forward

Before TB funding or treatment resources are allocated for diabetes care, more research is needed to demonstrate unequivocally that controlling diabetes can reliably improve TB outcomes. Studies examining the effect of diabetes primary prevention and treatment on TB incidence and outcomes would be particularly helpful in informing how diabetes care could best be coordinated with existing TB programs, and in deciding if TB funding should be used for this purpose. In addition, cost-effectiveness studies will be necessary to determine if the scarce funding available for global TB efforts should be used for costly diabetes care. Perhaps further highlighting the link between these two diseases will help boost international fundraising efforts for both.

Diabetes and TB represent a critical intersection between communicable and non-communicable diseases in some of the world's poorest countries. As the prevalence of NCDs continues to rise, the effects of NCDs on the prevention and treatment of infectious diseases will likely become more evident. The proven detrimental effect of diabetes on TB incidence and outcomes raises some important questions about whether TB funding and infrastructure should be used for diabetes treatment in developing countries, while also revealing numerous opportunities for collaboration and progress in both patient care and research. The recent report on diabetes and TB from the WHO and The International Union Against Tuberculosis and Lung Disease indicates that international interest in this topic is rising. Even if the management of these complex illnesses cannot be readily integrated, it is clear that the growing burden of diabetes and its effect on TB in developing countries should not be ignored.

## Abbreviations

DOTS: Directly Observed Therapy – Short Course
NCD: non-communicable disease
TB: tuberculosis
WHO: World Health Organization
